# A checklist of the millipedes of Georgia, Caucasus (Diplopoda)

**DOI:** 10.3897/zookeys.741.20042

**Published:** 2018-03-07

**Authors:** Mzia S. Kokhia, Sergei I. Golovatch

**Affiliations:** 1 Institute of Zoology, Ilia State University, K. Cholokashvili Ave., 3/5, Tbilisi 0162, Georgia; 2 Institute for Problems of Ecology and Evolution, Russian Academy of Sciences, Leninsky prospekt 33, Moscow 119071, Russia

**Keywords:** Colchis, distribution, endemism, fauna, Myriapoda

## Abstract

The diplopod fauna of Georgia, Transcaucasia, is very rich given the country’s relatively small territory, presently comprising 95 species from 42 genera, 12 families, and seven orders. Most of the Diplopoda known from Georgia are subendemics (39 species, or 38%), shared with one or more neighbouring countries, but another 33 species (33%) are strict endemics, nearly all highly localized, including 12 presumed troglobites. Several genera are likewise endemic to Georgia, including a few troglobionts. Within Georgia, the fauna of the western part (= Colchis) is particularly rich and diverse, the faunas of the central and eastern parts of the country growing increasingly depauperate inland and apparently following a rather gradual climatic aridisation gradient from west (the Black Sea coast) to east (Armenia and Azerbaijan). Much more work to include alpine and cave environments is required in order to reveal and refine the real diversity of Georgia’s Diplopoda.

## Introduction

**Figure 1.**
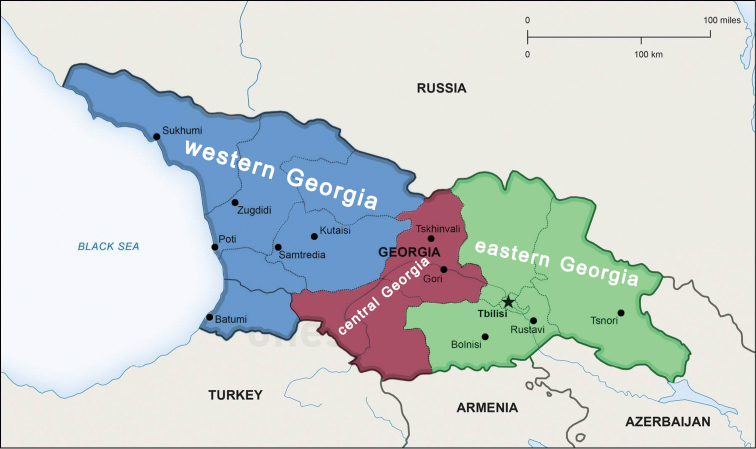
Crude geographical division of Georgia.

Georgia is one of the main countries in the Caucasus, lying between western Asia and Eastern Europe. It is bounded to the west by the Black Sea, to the north by Russia, to the south by Turkey, and to the southeast and east by Armenia and Azerbaijan (Fig. 1). The area is largely montane to high montane, situated between latitudes 41° and 44°N, and longitudes 40° and 47°E. The Greater Caucasus Mountain Range, or Caucasus Major, forms the northern border of Georgia, while the southern border is bounded by the Lesser Caucasus Mountains, or Caucasus Minor. The Caucasus Major is much higher in elevation (up to more than 5,000 m a.s.l.) than the plateau-like Caucasus Minor, both being connected by the submeridional Surami (= Likhi) Mountain Range which divides Georgia into the western and central + eastern parts. Both parts are quite varied in climate and biota. Western Georgia’s landscape ranges from lowland marsh-forests, swamps, and temperate rainforests within the Colchis Plain to eternal snows and glaciers, while the eastern part of the country even contains a small segment of semi-arid plains. Forests cover around 40% of Georgia’s territory, while the alpine/subalpine zone accounts for approximately 10% of the land. The climate of Georgia is extremely diverse, but largely mild to warm, considering the nation’s small size. There are two main climatic zones, roughly corresponding to the eastern and western parts of the country. The Greater Caucasus Mountain Range plays an important role in moderating Georgia’s climate and protects the nation from the penetration of colder air masses from the north. The Lesser Caucasus Mountains partially protect the region from the influence of dry and hot air masses from the south (Bondyrev et al. 2015).

The history of diplopodological research in the Caucasus generally, and in Georgia in particular, started with the works of Victor (1839), Brandt (1840) and Karsch (1881), followed by faunistic contributions by Timotheew (1897), Attems (1898, 1899, 1901, 1903, 1907), Lignau (1903, 1907, 1911, 1915, 1924), Muralewicz (1907, 1911, 1913, 1927) and Issaev (1911). Muralewicz (1911) was the first to thoroughly review the fauna of Caucasian Myriapoda known to that date. Verhoeff (1921, 1930), Jawłowski (1929) and Lohmander (1928, 1932) had added a few more species of Caucasian millipedes before a real milestone synthesis appeared. That historical stage culminated with Lohmander’s (1936) monograph which still serves, however outdated taxonomically, as one of the main sources of our knowledge of the Diplopoda of the Caucasus.

Several checklists, partly containing new faunistic records of Caucasian Diplopoda, appeared since then (Lang 1959, Kobakhidze 1964, 1965, Lokšina and Golovatch 1979, Talikadze 1984), but marked progress in the taxonomic study of millipedes in the region resumed only with contributions by Golovatch (1975, 1976a, 1976b, 1976c, 1977, 1979, 1980, 1981a, 1981b, 1984/85). Sporadic descriptions have since been upgraded to regional reviews of certain higher taxa such as genera, families and orders, with few exceptions only. These reviews mostly covered not only the Caucasus proper, but also the faunas of the adjacent parts of Turkey and Iran, e.g. the families Blaniulidae (Enghoff 1984, 1990, Golovatch and Enghoff 1990), Nemasomatidae (Enghoff 1985) and several tribes and genera of Julidae (Read 1992, Enghoff 1995, Evsyukov 2016b, Vagalinski and Lazányi 2018), all from the order Julida. The order Glomerida (Golovatch 1989a, 1989b, 1990, 1993, Golovatch and Chumachenko 2013), three orders of the subterclass Colobognatha (Golovatch et al. 2015, Zuev 2017), as well as the orders Polyxenida (Short 2015, Short et al. 2018), Polydesmida (Golovatch et al. 2016, Evsyukov et al. 2016) and Chordeumatida (Antić and Makarov 2016) have also been revised in the scope of the entire Caucasian fauna, sometimes even broader. The faunas of two larger areas in Ciscaucasia have also been reviewed and updated (Evsyukov and Golovatch 2013, Evsyukov 2016a, Zuev 2014). A couple of nature reserves at the Black Sea coast of the Russian Caucasus have likewise been thoroughly surveyed for their local millipede faunas, with some data on ecology and distribution (Chumachenko 2016, Korobushkin et al. 2016).

The present paper provides an up-to-date checklist (Table 1) of the millipede fauna of Georgia, based on all available publications. It shows very considerable progress achieved since the latest lists by Kobakhidze (1964, 1965), who grossly repeated Lohmander’s (1936) and added many new faunistic records, and by Talikadze (1984), who only considered the Colchidian part of the Caucasus, including the Black Sea coast area of Russia. Cave fauna has been reviewed within the entire former Soviet Union, including the Diplopoda of the Caucasus together with Georgia (Turbanov et al. 2016).

Georgia is conventionally divisible into three main parts: western, central, and eastern (Figure 1). This division is followed in the checklist below. Data are also given on the presence or absence of relevant species in the immediately neighbouring countries, including the Crimean Peninsula, as well as the distribution patterns and main literature sources. The checklist is arranged in alphabetic order per family, omitting subgeneric categories. All accepted designations are explained at the bottom of Table 1.

## Results

**Table 1.** Checklist of the Diplopoda of Georgia, with data on species distributions, both within and beyond the country, their statuses, and the main relevant literature sources. Dp = Distribution pattern.

Taxonomic composition	G	R	T	Ar	Az	Cr	St	Dp	Main relevant references
Class Diplopoda									
Order Polyxenida Family Polyxenidae Genus *Polyxenus* Latreille, 1803									
1. *Polyxenus argentifer* Verhoeff, 1921	G	+		+	+	+		AM	Short et al. 2018
2. *P. lagurus* (Linnaeus, 1758)	W, E					+		sc	Short et al. 2018
Family Lophoproctidae Genus *Lophoproctus* Pocock, 1894									
3. *Lophoproctus coecus* Pocock, 1894	G	+				+		EM	Short 2015, Short et al. 2018
Order Polyzoniida Family Hirudisomatidae Genus *Hirudiosoma* Fanzago, 1881									
4. *Hirudisoma roseum* (Victor, 1839)	G	+	+		+		se	EM	Golovatch et al. 2015
Order Siphonocryptida Family Siphonocryptidae Genus *Hirudicryptus* Enghoff & Golovatch, 1985									
5. *Hirudicryptus abchasicus* Golovatch, Esvyukov & Reip, 2015	W	+					se	Ca	Golovatch et al. 2015, Zuev 2017
Order Glomerida Family Glomeridae Genus *Hyleoglomeris* Verhoeff, 1910									
6. *Hyleoglomeris awchasica* (Brandt, 1840)	W	+					se	Ca	Golovatch 1976a, 1989b
7. *H. specialis* Golovatch, 1989	E	+					se	Ca	Golovatch 1989b
Genus *Trachysphaera* Heller, 1858									
8. *Trachyspaera costata* (Waga, 1857)	G	+	+	+	+	+		EuM	Golovatch 1990, 2008
9. *T. fragilis* Golovatch, 1976	G						t, e	Ca	Golovatch 1990, Golovatch and Turbanov 2017
10. *T. minuta* Golovatch, 1976	G	+	+	+			se	Ca	Golovatch 1990
11. *T. orientalis* Golovatch, 1976	W						t, e	Ca	Golovatch 1976c, 1990
12. *T. radiosa* (Lignau, 1911)	W	+					se	Ca	Golovatch 1976c, 1990
13. *T. solida* Golovatch, 1976	W, C						se	Ca	Golovatch 1976c, 1990, 1993
Family Glomeridellidae Genus *Typhloglomeris* Verhoeff, 1898									
14. *Typhloglomeris lohmanderi* (Golovatch, 1989)	C, E	+		+			se	Ca	Golovatch 1989a, 2003
Order Chordeumatida Family Anthroleucosomatidae Genus *Acanthophorella* Antić & Makarov, 2016									
15. *Acanthophorella barjadzei* Antić & Makarov, 2016	W						t, e	Ca	Antić and Makarov 2016
Genus *Adshardicus* Golovatch, 1981									
16. *Adshardicus strasseri* Golovatch, 1981	W		+				se	Ca	Enghoff 2006, Antić and Makarov 2016
Genus *Alpinella* Antić & Makarov, 2016									
17. *Alpinella waltheri* Antić & Makarov, 2016	E						e	Ca	Antić and Makarov 2016
Genus *Brachychaetosoma* Antić & Makarov, 2016									
18. *Brachychaetosoma turbanovi* Antić & Makarov, 2016	W						t, e	Ca	Antić and Makarov 2016
Genus *Caucaseuma* Strasser, 1970									
19. *Caucaseuma kelasuri* Antić & Makarov, 2016	W						e	Ca	Antić and Makarov 2016
20. *C. variabile* Antić & Makarov, 2016	C	+					se	Ca	Antić and Makarov 2016
Genus *Cryptacanthophorella* Antić & Makarov, 2016									
21. *Cryptacanthophorella manubriata* Antić & Makarov, 2016	W, C						e	Ca	Antić and Makarov 2016
Genus *Dentatosoma* Antić & Makarov, 2016									
22. *Dentatosoma denticulatum* Antić & Makarov, 2016	W						e	Ca	Antić and Makarov 2016
23. *D. magnum* Antić & Makarov, 2016	W	+					se	Ca	Antić and Makarov 2016
24. *D. zeraboseli* Antić & Makarov, 2016	W						e	Ca	Antić and Makarov 2016
Genus *Georgiosoma* Antić & Makarov, 2016									
25. *Georgiosoma bicornutum* Antić & Makarov, 2016	W						t, e	Ca	Antić and Makarov 2016
Genus *Herculina* Antić & Makarov, 2016									
26. *Herculina oligosagittae* Antić & Makarov, 2016	W						e	Ca	Antić and Makarov 2016
27. *H. polysagittae* Antić & Makarov, 2016	C						e	Ca	Antić and Makarov 2016
Genus *Heterocaucaseuma* Antić & Makarov, 2016									
28. *Heterocaucaseuma longicorne* Antić & Makarov, 2016	W						t, e	Ca	Antić and Makarov 2016
29. *Heterocaucaseuma mauriesi* (Golovatch & Makarov, 2011)	W						t, e	Ca	Golovatch and Makarov 2011, Antić and Makarov 2016
Genus *Metamastigophorophyllon* Ceuca, 1976									
30. *Metamastigophorophyllon giljarovi* (Lang, 1959)	W	+					se	Ca	Antić and Makarov 2016
31. *M. hamatum* Antić & Makarov, 2016	W	+					se	Ca	Antić and Makarov 2016
32. *M. lamellohirsutum* Antić & Makarov, 2016	W						e	Ca	Antić and Makarov 2016
33. *M. torsivum* Antić & Makarov, 2016	G				+		se	Ca	Antić and Makarov 2016
Genus *Paranotosoma* Antić & Makarov, 2016									
34. *Paranotosoma attemsi* Antić & Makarov, 2016	W						e	Ca	Antić and Makarov 2016
35. *P. cordatum* Antić & Makarov, 2016	W						e	Ca	Antić and Makarov 2016
36. *P. subrotundatum* Antić & Makarov, 2016	W	+					se	Ca	Antić and Makarov 2016
Genus *Pseudoflagellophorella* Antić & Makarov, 2016									
37. *Pseudoflagellophorella eskovi* Antić & Makarov, 2016	C, E			+	+		se	Ca	Antić and Makarov 2016
38. *P. mirabilis* Antić & Makarov, 2016	W						e	Ca	Antić and Makarov 2016
39. *P. papilioformis* Antić & Makarov, 2016	E				+		se	Ca	Antić and Makarov 2016
Genus *Ratcheuma* Golovatch, 1985									
40. *Ratcheuma excorne* Golovatch, 1985	W						t, e	Ca	Golovatch 1984/85, Antić and Makarov 2016
Order Julida Family Blaniulidae Genus *Cibiniulus* Verhoeff, 1927									
41. *Cibiniulus phlepsii* (Verhoeff, 1897)	W		+					EuM	Enghoff 1984, 2006
Genus *Nopoiulus* Menge, 1851									
42. *Nopoiulus brevipilosus* Enghoff, 1984	W						t, e	Ca	Enghoff 1984
43. *N. densepilosus* Enghoff, 1984*	W				+			Ca	Enghoff 1984, Golovatch and Enghoff 1990
44. *N. golovatchi* Enghoff, 1984	W		+					Ca	Enghoff 1984, 1990
45. *N. kochii* (Gervais, 1847)	G	+	+	+	+			sc	Enghoff 1984, Golovatch and Enghoff 1990
Family Nemasomatidae Genus *Nemasoma* C.L. Koch, 1847									
46. *Nemasoma caucasicum* (Lohmander, 1932)	G	+	+	+	+		se	Ca	Enghoff 1985
Family Julidae Genus *Amblyiulus* Silvestri, 1896									
47. *Amblyiulus adsharicus* Lohmander, 1936	W						e	Ca	Lohmander 1936
48. *A. georgicus* Lohmander, 1932	C						e	Ca	Lohmander 1932
Genus *Archileucogeorgia* Lohmander, 1936									
49. *Archileucogeorgia abchasica* Lohmander, 1936	W						t, e	Ca	Lohmander 1936
50. *Archileucogeorgia satunini* Lohmander, 1936	W						e	Ca	Lohmander 1936
Genus *Brachyiulus* Berlese, 1884									
51. *Brachyiulus lusitanus* Verhoeff, 1898`	C				+			M	Lohmander 1936
Genus *Catamicrophyllum* Verhoeff, 1900									
52. *Catamicrophyllum caucasicum* (Attems, 1901)	G	+	+	+			se	Ca	Lohmander 1936, Enghoff 1995
Genus *Calyptophyllum* Brolemann, 1922									
53. *Calyptophyllum* sp.	W						?	?	Lohmander 1936, Enghoff 1995
Genus *Chaetoleptophyllum* Verhoeff, 1898									
54. *Chaetoleptophyllum flexum* Golovatch, 1979	G	+					se	Ca	Golovatch 1979, Chumachenko 2016, Korobushkin et al. 2016
Genus *Cylindroiulus* Verhoeff, 1894									
55. *Cylindroiulus bellus* (Lignau, 1903)	W?	+				+		EM	Lignau 1903, Read 1992, Chumachenko 2016
56. *C. crassiphylacum* Read, 1992	G		+				se	Ca	Read 1992
57. *C. kacheticus* Lohmander, 1936	E	+					se	Ca	Read 1992
58. *C. olgainna* Read, 1992	W						e	Ca	Read 1992
59. *C. parvus* Lohmander, 1928	C, E				+		se	Ca	Read 1992
60. *C. placidus* (Lignau, 1903)	W, C	+					se	Ca	Read 1992
61 *C. pterophylacum* Read, 1992	W, C	+					se	Ca	Read 1992, Zuev 2014
62. *C. quadrus* Read, 1992	G	+					se	Ca	Read 1992
63. *C. ruber* (Lignau, 1903)	W	+					se	Ca	Read 1992
64. *C. schestoperovi* Lohmander, 1936	W	+					se	Ca	Lohmander 1936, Read 1992
65. *C. truncorum* (Silvestri, 1896)	W							sc	Read 1992
Genus *Grusiniulus* Lohmander, 1936									
66. *Grusiniulus redikorzevi* Lohmander, 1932	C						e	Ca	Lohmander 1936, Vagalinski and Lazányi 2018
Genus *Julus* Linnaeus, 1758									
67. *Julus colchicus* Lohmander, 1936	W	+	+				se	Ca	Lohmander 1936, Enghoff 2006
68. *J. kubanus* Verhoeff, 1921	W	+					se	Ca	Lohmander 1936, Kobakhidze 1965
69. *J. lindholmi* Lohmander, 1936	W						e	Ca	Lohmander 1936
Genus *Leptoiulus* Verhoeff, 1894									
70. *Leptoiulus disparatus* Lohmander, 1936	C		+				se	Ca	Lohmander 1936, Enghoff 2006
71. *L. tanymorphus* (Attems, 1901)	C, E				+			Ca	Lohmander 1936
Genus *Leucogeorgia* Verhoeff, 1930									
72. *Leucogeorgia longipes* Verhoeff, 1930	W						t, e		Verhoeff 1930
73. *L. rediviva* Golovatch, 1983	W						t, e	Ca	Golovatch 1983
Genus *Megaphyllum* Verhoeff, 1894									
74. *Megaphyllum dioscoriadis* (Lignau, 1915)	W	+					e	Ca	Lignau 1915, Lohmander 1936, Chumachenko 2016, Vagalinski and Lazányi 2018
75. *M. hercules* (Verhoeff, 1901)	W	+						EM	Lazányi and Vagalinski 2013
76. *M. spathulatum* (Lohmander, 1936)	W?	?						Ca	Lohmander 1936, Lazányi and Vagalinski 2013
Genus *Omobrachyiulus* Lohmander, 1936									
77. *Omobrachyiulus adsharicus* (Lohmander, 1936)	W						e	Ca	Lohmander 1936, Vagalinski and Lazányi 2018
78. *O. brachyurus* (Attems, 1899)	G	+	+	+	+			EM	Lohmander 1936, Enghoff 2006, Vagalinski and Lazányi 2018
79. *O. curvocaudatus* (Lignau, 1903)	W	+					se	Ca	Lohmander 1936, Vagalinski and Lazányi 2018
80. *O. divaricatus* (Lohmander, 1936)	G			+			se	Ca	Lohmander 1936, Vagalinski and Lazányi 2018
81. *O. hortensis* (Golovatch, 1981)	W						e	Ca	Golovatch 1981, Vagalinski and Lazányi 2018
82. *O. implicitus* Lohmander, 1936 (= *O. i. ritsensis* (Golovatch, 1981))	W	+					se	Ca	Lohmander 1936, Chumachenko 2016, Vagalinski and Lazányi 2018
83. *O. macrourus* (Lohmander, 1928) (= *O. m. abchasicus* (Lohmander, 1936))	W, C						e	Ca	Lohmander 1936, Kobakhidze 1965, Vagalinski and Lazányi 2018
Genus *Pachyiulus* Berlese, 1883									
84. *Pachyiulus flavipes* (C.L. Koch, 1847)	W					+		M	Lohmander 1936
85. *Pachyiulus krivolutskyi* Golovatch, 1977	W	+					se	Ca	Evsyukov 2016
Order Polydesmida Family Paradoxosomatidae Genus *Oxidus* Cook, 1911									
86. *Oxidus gracilis* (C.L. Koch, 1847)	W	+						sc	Lignau 1915, Lohmander 1936, Chumachenko 2016
Family Polydesmidae Genus *Brachydesmus* Heller, 1858									
87. *Brachydesmus assimilis* Lohmander, 1936	C, E	+					se	Ca	Golovatch et al. 2016
88. *B. furcatus* Lohmander, 1936	W	+					se	Ca	Golovatch et al. 2016
89. *B. kalischewskyi* Lignau, 1915	G	+	+	+	+		se	Ca	Golovatch et al. 2016
90. *B. kvavadzei* Golovatch, Evsyukov & Reip, 2016	W						e	Ca	Golovatch et al. 2016
91. *B. simplex* Golovatch, Evsyukov & Reip, 2016	W	+					se	Ca	Golovatch et al. 2016
92. *B. superus* Latzel, 1884	W	+						sc	Golovatch et al. 2016
Genus *Polydesmus* Latreille, 1803									
93. *Polydesmus abchasius* Attems, 1899	W	+					se	Ca	Golovatch et al. 2016
94. *P. lignaui* Lohmander, 1936	W	+					se	Ca	Golovatch et al. 2016
95. *P. mediterraneus* Daday, 1889	W					+		EM	Golovatch et al. 2016

**Designations**: G – entire Georgia; W – western Georgia; C – central Georgia; E – eastern Georgia; R – Russian Caucasus; T – Turkey; Ar – Armenia; Az – Azerbaijan; Cr – Crimean Peninsula; (+) – present; St – status; e – endemic to Georgia; se – subendemic to Georgia; t – presumed troglobiont; sc – subcosmopolitan; AM – Ancient Mediterranean; EuM – Euro-Mediterranean; M – Mediterranean; EM – eastern Mediterranean; EE – eastern European; Ca – Caucasian.

## Discussion

As is evident from the above list, the millipede fauna of Georgia is, surprisingly, very diverse, especially so given the relatively small territory it covers. This is hardly surprising, because Diplopoda are largely mesophilous forest-dwellers (e.g., Kime and Golovatch 2000, Golovatch and Kime 2009). Georgia with its mostly mild climate and large woodland areas supports the richest millipede fauna in the entire Caucasus, nearly twice as rich as neighbouring Azerbaijan (Bababekova 1996, a quite poorly compiled list) or Iran (Enghoff and Moravvej 2005), and approximately 2/3 as diverse as the fauna of the so much larger Turkey (Enghoff 2006, 135 species), for all of which rather modern country checklists are available. Although the bulk of the fauna of Georgia is represented by epigean taxa, the abundant limestone massifs, primarily those lying at the northern and northeastern peripheries of the Colchis, harbour numerous karstic caves with their own fauna. Troglobionts do account for a considerable proportion (12 species, or 14%) of Georgia’s millipede species (Barjadze et al. 2015, Turbanov et al. 2016).

Western Georgia, the Colchis (Fig. 1), is especially rich in millipedes, apparently due to the moist and mild climate near the warm Black Sea, highly varied, but largely forested habitats, and abundant karst caves. The Colchidian millipede fauna is also the richest in endemics, both at the species and generic levels. The orders Chordeumatida and Julida are particularly strongly diversified in Georgia. As well the country supports also *Hirudicryptus abchasicus*, a subendemic representing one of the most relict diplopod orders, Siphonocryptida, which presently comprises only seven species in two genera and a single family (Golovatch et al. 2015, Zuev 2017). Central and eastern parts of Georgia are increasingly drier, in places even semi-arid, and the millipede fauna generally demonstrates a decline in diversity from the Black Sea coast inland, appearing to follow rather gradual climatic aridisation gradient from west to east.

Most of the Diplopoda known from Georgia are subendemics (40 species, or 42%), shared with one or more neighbouring countries, but another 33 species (34%) are strict endemics, nearly all highly localized, including 12 presumed troglobites. Several genera are likewise endemic to Georgia, including a few troglobionts. The proportions of the remaining, more widely distributed, species are rather modest, represented by Mediterranean, Euro-Mediterranean, eastern Mediterranean, eastern European or ubiquitous elements, but even among the latter the subcosmopolitan *Nopoiulus kochii* may have originated in the Caucasus, because the remaining congeners (from all subgenera) seem to be endemic to the Caucasus region (Golovatch and Enghoff 1990).

The present checklist must be understood as temporary, far from complete, marking only the state of knowledge of diplopodological research in Georgia. Several of Lohmander’s *nomina nuda* listed by Kobakhidze (1964) are thereby omitted. Much more work is required to reveal the real diversity of Georgia’s Diplopoda. Discoveries and descriptions of numerous new taxa, both species and probably even genera, can still be expected in the future. Further faunistic records are necessary to refine not only the taxonomy and the above list, but also the distributions, both horizontal and vertical. Very little is known yet concerning high-montane millipedes, in particular, whether strictly alpine Caucasian/Georgian endemics exist at all, like those few recorded from the Pyrenees and Alps. Finally, cave explorations in Georgia will undoubtedly reveal many more new troglobionts, including diplopods.
